# Evaluation of the Impact of Noise Pollution on the Workers of the Bottling Line of the Limited Company of Breweries of Guinea (SOBRAGUI)

**DOI:** 10.7759/cureus.18033

**Published:** 2021-09-16

**Authors:** Alpha Oumar Diallo, Carlos Othon Guelngar, Mamadou Baillo Balde, Omar Issa Omar, Alimou Sinayoko, Amadou Lamarana Diallo, Mouhammad Bah

**Affiliations:** 1 Ear Nose Throat (ENT) & Cervico-Facial Surgery (CFS) Department, Ignace Deen National Hospital, Conakry, GIN; 2 Neurology Department, Ignace Deen National Hospital, Conakry, GIN; 3 Ear Nose Throat (ENT) & Cervico-Facial Surgery (CFS) Department, Donka National Hospital, Conakry, GIN

**Keywords:** noise pollution, worker, sobragui, conakry, evaluation

## Abstract

Introduction

Noise or noise pollution is a phenomenon that causes an auditory sensation considered unpleasant, undesirable, and annoying, which may present a danger to health in general and the auditory system in particular. The objective of this study was to evaluate the impact of noise pollution among workers in a brewery in the city of Conakry.

Patient and methods

This is a prospective descriptive study, of two months' duration (November 11, 2019, to January 10, 2020) carried out in the industrial unit of the Limited Company of Breweries of Guinea (SOBRAGUI). We included all the workers of the company exposed to noise pollution in their workplaces, after their informed consent.

Results

The age of the workers varied between 22 and 70 years, with a mean age of 44.14 ± 8.07 years. We noted a male predominance of 99.22% (n=128) cases. The average duration of exposure was 14.28 ± 6.78 years, with variations ranging from one year to 35 years. Among the auditory disorders, we noted tinnitus in 63.8% (n=74), hypoacusis in 56.9% (n=66), and auditory fatigue in 52.6% (n=61) of cases. These signs were often associated with each other and with other extra-auditory symptoms in the same worker. Audiometry revealed a moderate to severe hearing loss in 13.8% (n=16) and profound deafness in 0.9% (n=1) of cases. The impact of noise pollution on workers' performance was reflected in concentration difficulties in 57.7% (n=67) of cases.

Conclusion

Noise pollution is present in the SOBRAGUI bottling line. It has a negative impact on the health of workers and alters their work performance.

## Introduction

Noise or noise pollution is a phenomenon that causes an auditory sensation that is considered unpleasant, undesirable, and annoying, and that may present a danger to health in general and the auditory system in particular [1]. According to the WHO, noise exposure of more than 85 dB for eight hours a day at work or more than 100 dB for 15 minutes is harmful to the health of workers [2]. In France, noise pollution has been recognized as an occupational disease since 1963 [3]. It represents an important risk in the workplace that affects many economic sectors and other professional activities. The high risk of occupational accidents related to noise pollution is related to its masking effect on warning signals, thus disrupting verbal communication and diverting the attention of workers. It is responsible for approximately 37% of the causes of hearing loss in adults and remains an important factor in employment-related morbidity internationally [4]. This ultimately leads to reduced performance and lower productivity [5-6].

In Canada, a study of the impact of hearing conservation programs on the incidence of noise-induced hearing loss in 22,376 workers had shown a threshold shift of 10 dB or more in at least 2,839 of them [7].

In France, a medical risk surveillance survey in 2010 had reported that long-term noise exposure (more than 20 hours per week) at high levels (≥85 dB/(A)) affected 4.8% of employees, of which the sectors most concerned were industry (16.8%) and construction (10.5%) [3].

In Algeria, Hammoudi N et al., in a study on the relationship between noise and blood pressure in an airport environment, had reported hypertension in 9.25% of ground staff and 16.63% of aircrew [8].

In Tunisia, Brahem A et al. had shown in 2019 that, after exposure to a sound level varying between 75 dB and 103 dB for eight hours, almost all the employees of an electricity company had declared irritability, nervousness, and ringing in the ears [9].

In Côte d'Ivoire, Yeboué-Kouamé BY et al., in a study on the assessment of noise risk at 881 workstations in 2018, had revealed that 60% of the workstations assessed exposed employees to more than 85 dB(A) and 33.5% to more than 90 dB(A) over a working time of more than two hours daily [10].

In Guinea, very little data are available on occupational risks related to noise pollution in companies, and the regulations on noise in the workplace remain insufficient. This represents a major handicap for social security and occupational risk prevention organizations. The objective of this study was therefore to evaluate the impact of noise pollution on workers in the bottling line of a brewery in Conakry.

## Materials and methods

This is a cross-sectional, descriptive, two-month study (November 11, 2019, to January 10, 2020), carried out in the industrial unit of the Limited Company of Breweries of Guinea (SOBRAGUI). We included in this study all workers of all ages and genders working in the bottling line of the said company and who gave their informed consent. We administered a pre-established questionnaire to each of the workers staying temporarily or for a long time (between eight and 10 hours per day) at the bottling line where dosimetry had shown variable sound exposure thresholds between 85 and 95 dB (A). Systematic luminaire tonal audiometry was carried out at least 14 hours after the last exposure to noise in the workplace using a GSI 18 audiometer, meeting the ISO 11690-1.2 1996 standard. Audiometric tests were carried out in a soundproof cabin. Our data were captured using Kobocollect and analyzed using SPSS 21.0 software (IBM Corp., Armonk, NY). The survey was conducted with strict respect for anonymity and moral and physical integrity while keeping the confidentiality of the study. This study was reviewed and approved by the ethics committee of the Faculty of Health Sciences and Techniques of the University of Conakry.

## Results

Of the 350 workers at the Guinean Brewery Company (SOBRAGUI), 129 workers were interested in the investigation because they worked at the bottling line, i.e., 36.86% of cases. The age of the workers varied between 22 and 70 years with an average age of 44.14 ± 8.07 years. The 40 to 49 age group is the most represented, at 47.3% (n = 61) of cases. We noted a male predominance of 99.22% (n = 128) of the cases. Ninety-two (92) respondents, i.e., 71.3%, worked in the production sector, which includes the brewing and packaging units that are the most exposed to noise pollution with an average duration of exposure of 14.28 ± 6.78 years and extremes: 01 year and 35 years (Table 1).

**Table 1:** Distribution of workers according to socio-demographic characteristics Average age: 44.14 ± 8.07 years; Extreme: 22 years and 70 years Average exposure time: 14.28 ± 6.78 years; Extreme: 1 year and 35 years

Socio-demographic characteristics	Effective	Percentage
Age		
20 – 29	3	2.3
30 – 39	35	27.1
40 – 49	61	47.3
≥ 50	30	23.3
Sex		
Male	128	92.2
Female	1	0.8
Workplace		
Production	92	71.3
Maintenance	31	24.0
Forklift operator	6	4.7
Noise exposure time		
0 to 10	36	27.9
11 to 20	71	55.0
21 to 30	20	15.5
> 30	2	1.6
Total	129	100

**Figure 1:**
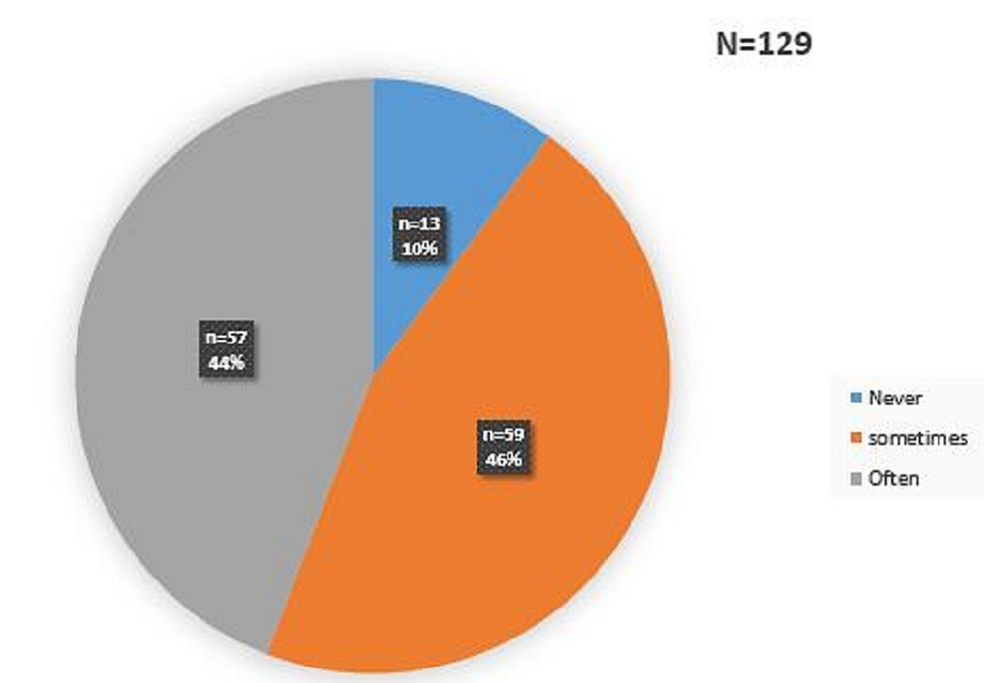
Distribution of workers according to the discomfort felt in the workplace

During questioning, only 13 workers, or 10% of cases, said they had never felt discomfort related to noise pollution in the workplace. The 116 other workers had sometimes or often felt discomfort, which could be auditory or extra-auditory but related to the noise nuisance (Figure 1). 

**Figure 2:**
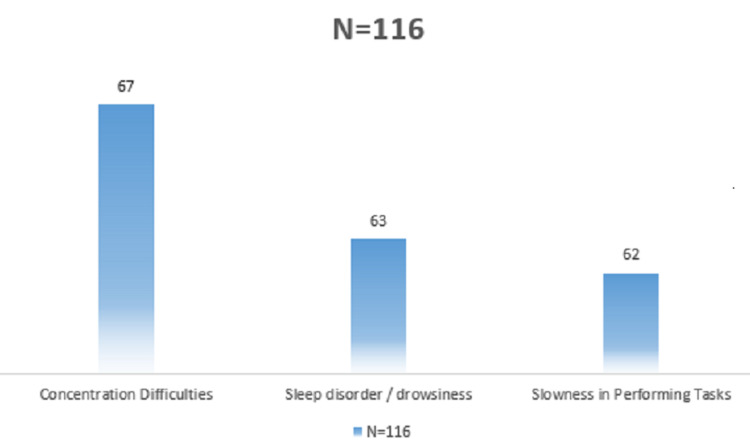
Frequency of the impact of noise pollution on the quality of work

Among the hearing problems, we noted tinnitus in 63.8% (n = 74), hearing loss in 56.9% (n = 66) and hearing fatigue in 52.6% (n = 61) of cases. These signs were often associated with each other and with other extra-auditory symptoms in the same worker. All respondents were tested with an audiometer which revealed moderate to severe sensorineural hearing impairment in 13.8% (n = 16) and one case of profound sensorineural hearing loss (Table 2). The repercussion of noise pollution on workers' performance was evident and resulted in difficulty concentrating in 57.7% (n = 67) of cases (Figure 2).

**Table 2:** Distribution of workers according to clinical characteristics due to noise pollution (n = 116)

Clinical characteristics	Effective	Percentage
Symptoms		
Tinnitus	74	63.8
Hearing loss	66	56.9
Hearing fatigue	61	52.6
Irritability	52	44.8
Chronic headache	52	44.8
Palpitation	51	43.9
Degree of deafness		
Mild deafness	99	85.3
Moderate deafness	13	11.2
Severe deafness	3	2.6
Profound sensorineural hearing loss	1	0.9
Site of lesion		
Bilateral	64	55.2
One-sided	52	44.8
Total	116	100

## Discussion

Noise pollution is one of the pollutants or factors that threaten health in an industrial environment. It impairs the quality of life of workers and disrupts their socio-professional integration. Although many other adverse effects of exposure to high noise levels have been reported, noise-induced hearing loss is recognized as the main and most direct effect on the health of exposed workers. It is the most common occupational disease. It is estimated that 80% of people with occupational hearing loss live in low- and middle-income countries and that almost half of the industrial workforce is exposed to hazardous occupational noise. Ninety percent (90%) work in areas where noise levels exceed 85 decibels on average [11].

The workers in the bottling line of the Société Anonyme des Brasseries de Guinée are mostly adults with a mean age of 44.14 ± 8.07 years. Hinson AV et al., in Benin, report an average age of 35.6 ± 10.4 years with extremes of 17 and 68 years [12]. This result could be explained by the fact that this is a long-established industrial unit in a country that still trusts its experienced staff. The male predominance was similar to the study by Amel AE et al. in 2016 [13]. This confirms the fact that in our developing countries, men are usually recruited for production or handling tasks that are considered labor-intensive.

Despite the correct use of protective equipment, the exposure time of our workers was particularly long. The majority spent eight and 10 hours in a noisy working environment, exposed to machine noise for several years. The same was true in the countries of the sub-region, with an average duration of exposure to noise of 11.2 years in Burkina Faso [14].

The impact of this noise nuisance was more or less marked in almost 90% of our respondents. This included tinnitus, hearing loss, and hearing fatigue. Oubian S et al., in a study on the impact of noise pollution on the quality of life of power plant workers in Burkina Faso, found 34% hearing loss [14]​​​​​​​. These hearing problems may be isolated or associated with other symptoms such as headaches, sleep disturbances, and irritability. These associated extra-auditory manifestations had a negative impact on the social life of the respondents [14].

All noise-exposed workers underwent an audiometric test showing some degree of hearing loss. This result would be comparable to that reported by Shahid A et al., in Pakistan, who reported 79% hearing loss greater than or equal to 25 dB loss [15]​​​​​​​. In fact, the long-term occupational noise exposure of bottling line workers will continue to have a significant influence on their hearing thresholds even after the noise exposure has ended [13]. In contrast to our survey, where most of the respondents had developed bilateral hearing loss, Sriopas A et al., in Thailand, found a predominance of unilateral hearing loss in 73.68% (n = 42) of the workers [16]​​​​​​​

The impact of noise pollution on workers' performance in the workplace was evident, resulting in decreased productivity and increased work-related accidents. In addition, social and family life was compromised for most of these workers due to noise pollution [14].

## Conclusions

Noise pollution is present at the SOBRAGUI bottling line. It negatively impacts the health of workers and affects their work performance. Measures to improve the working environment at the bottling line of this industrial unit must be put in place in order to reduce not only the threshold but also the time of exposure of workers to noise pollution.
